# MOLT: multi-object and lineage tracking in 2D and 3D biomedical time-series imaging

**DOI:** 10.1186/s12859-026-06434-y

**Published:** 2026-04-01

**Authors:** Ben Bausch, Mina Naseh, Goncalo Gaspar Alves, Andreas Husch, Thomas Gillet, Michael T. Heneka, Jorge Goncalves, Sergio Castro-Gomez, Shekoufeh Gorgi Zadeh

**Affiliations:** 1 University of Luxembourg, LCSB, 4362 Esch-sur-Alzette, Luxembourg; 2 Center for Neurology, Department of Parkinson, Sleep and Movement Disorders, University Hospital Bonn, University of Bonn, 53127 Bonn, Germany; 3 Former Association with the University of Luxembourg, LCSB, 4362 Esch-sur-Alzette, Luxembourg; 4 Department of Infectious Diseases and Immunology, University of Massachusetts Medical School, Worcester, MA 01655 USA; 5 Institute of Physiology II, University Hospital Bonn, University of Bonn, 53115 Bonn, Germany; 6 Department of Plant Sciences, University of Cambridge, Downing St, Cambridge, CB2 3EA UK

**Keywords:** Multi-object tracking, Lineage, 2D, 3D, Training-free, Cell tracking

## Abstract

Tracking object instances, such as individual living cells or molecular accumulations, and their behavior is a common challenge in 2D and 3D volumetric biomedical imaging. Combined with dynamic environments caused by unintentional camera misalignment over longitudinal studies, deformable tissues, and morphologically changing instances, object tracking poses a challenging and time-consuming task in biomedical settings. This paper presents a robust 2D and 3D object tracker designed to handle such scenarios, ensuring consistent identification of physical objects over time. Our method combines image registration with a novel graph-based conflict resolution algorithm for object and lineage tracking, handling camera misalignment and object movement. The algorithm accommodates morphological changes including splitting, merging, growing, shrinking, emerging, and vanishing object instances. This training-free framework serves as a post-processing step after instance segmentation, making it compatible with many tracking-by-detection approaches and generalist segmentation models such as CellPose-SAM. The proposed framework provides a flexible, training-free, and interpretable solution for long-term tracking in neuroscience, cell biology, and medical imaging. Our method is evaluated in two biomedical contexts: tracking 3D Beta-amyloid accumulations in in-vivo two-photon fluorescence imaging and monitoring cell movement and proliferation in 2D cultures. On 2D cell-tracking datasets, MOLT achieves TAR scores of (92.618–96.145%) on CellPose-SAM annotations, outperforming established TrackMate baselines on two of three datasets. For 3D Beta-amyloid plaque tracking, MOLT achieves a Lineage Reconstruction score (LNR) of 83.394%, demonstrating accurate lineage reconstruction under complex splitting and merging dynamics.

## Background

**Fig. 1:**
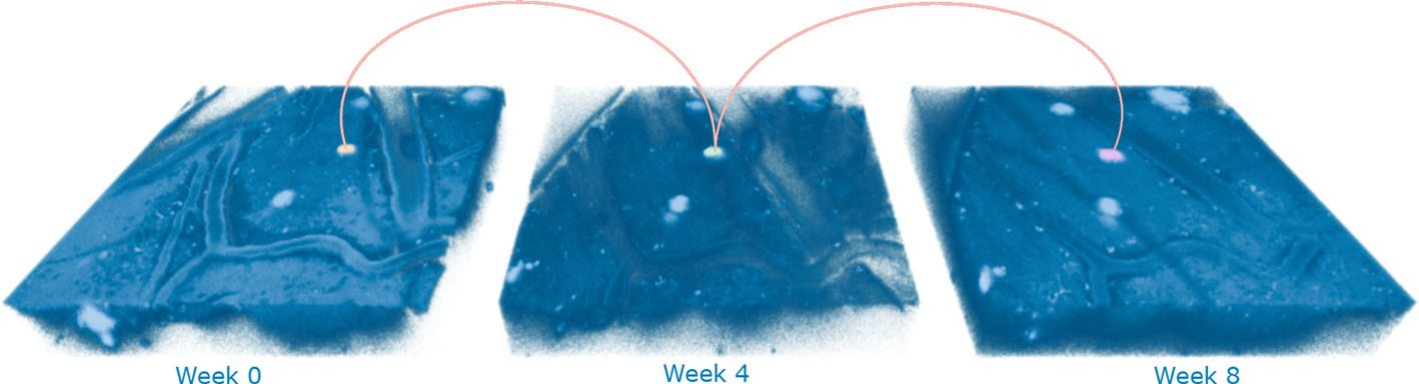
Tracking an individual Beta-amyloid accumulation (plaque) in volumetric photon fluorescence excitation microscopy over multiple weeks

Multi-object tracking (MOT) denotes the integrated process of detecting or segmenting multiple target objects within sequential imaging data while maintaining their temporal identities through consistent labeling. The inherent complexity of MOT is highly dependent on the application domain, each presenting distinct methodological challenges. In non-biomedical applications, such as autonomous driving [1], MOT systems must contend with highly dynamic scenes characterized by independently moving entities, partial or complete occlusions, and objects transitioning in and out of the field of view. These scenarios are typically recorded via high-resolution, mobile pinhole cameras, with temporal sampling rates sufficient to generate fine-grained trajectory data at multiple frames per second. Biomedical applications exhibit analogous challenges but introduce additional constraints, including non-rigid, deformable backgrounds due to tissue motion, as shown in Fig. 1. Moreover, the tracked objects can undergo morphological changes, such as growth, shrinkage, fission, or fusion, thereby necessitating the explicit modeling of lineage relationships to ensure accurate tracking. Due to the physical limitations of biomedical scanners and microscopes, the longitudinal imaging data, containing up to hundreds or thousands of individual objects, often suffer from a trade-off between spatial and temporal resolution.

A key distinction in biomedical tracking lies in the morphological dynamics of the objects being tracked. In cell tracking, biological constraints dictate that cells only arise from parent cells and never merge, requiring a framework that enforces strict lineage continuity. In contrast, protein aggregation tracking demands handling repeated merging, splitting, and spontaneous emergence, where the focus shifts to capturing all interactions over time. Most existing trackers are tailored to one of these scenarios and cannot be directly applied to the other. The primary contribution of this work is a novel graph-based conflict resolution algorithm that handles both paradigms within a single unified framework, distinguishing it from prior overlap-based trackers.

Offering a comprehensive analysis of state-of-the-art MOT methodologies in non-medical applications, [2] categorizes them into tracking-by-detection (TBD) and end-to-end (E2E) paradigms. TBD approaches adopt a sequential pipeline, where objects are first detected or segmented in each frame, followed by an association step to link detections across frames and establish temporally consistent trajectories. In contrast, E2E methods integrate detection and tracking into a unified framework, performing these tasks jointly rather than sequentially to enhance robustness and efficiency.

Splitting the tracking task into a two step procedure, TBD methods can use either traditional segmentation techniques (e.g., [3, 4]), domain-specific deep learning models [5–8], or current state-of-the-art generalist deep learning models [9]. The subsequent temporal association of the segmented structures can be performed using integer linear programming with euclidean distance or Intersection-over-Union as cost functions [10], segmentation overlap based frameworks [11] or deep learning models, such as graph neural networks [12] or transformers [13].

E2E methods frequently employ high-performing deep learning architectures to learn joint detection and tracking [14]. A notable limitation of many E2E methods is their inherent domain-specificity. The learned features often rely heavily on the modality of the time-series data (e.g., tracking of lesions in CT scans), making them less transferable to other modalities. While E2E methods can in principle be retrained for new domains, this requires sufficiently large datasets with ground truth annotations, which are frequently unavailable in biomedical research. For the protein aggregation dataset used in this work, no annotated ground truth existed prior to this study, making E2E retraining infeasible. TBD methods therefore remain a practical alternative in such settings, as they do not require annotated training data.

This work focuses on two multi-object tracking scenarios that are very common in biomedical imaging. The first scenario (S1) considers the tracking of objects where the relative positions in between the objects vary only slightly over time, while the whole region of interest can undergo strong movement from one measurement to the next. Common examples of such a scenario are the tracking of protein accumulations in deformable living tissue [15] and lesions tracking in computed tomography scans [16]. The second scenario (S2) allows for significant relative movement between the objects over time given a sufficiently high sampling frequency capturing the dynamics of the system, e.g. observing living cells in in-vitro microscopy videos. We propose an algorithm, that can handle both scenarios with only a single simple change in the configuration.

In both scenarios, the misalignment of the captured regions of interest (ROI) in consecutive measurements is a challenge when tracking multiple objects for several reasons. Misalignment can negatively impact the performance of overlap-based tracking methods and can additionally lead to objects moving outside the captured region of interest. Similar to our method, [17] uses affine registrations to adjust for the misalignment across multiple histological slices. They perform the registrations on 2D serial whole-slide microscopy images before performing multi-object tracking. This problem is common also in other fields where the camera and the region of interest move independent of each-other, such as aerial image processing where image registration is used to compensate for the movement of camera in aerial videos of landscapes for downstream object tracking [18]. While our method can be applied to such settings as well, the evaluation will be conducted on two biomedical applications in section Results: 2D cell tracking in 2D in-vitro microscopy videos and the in-vivo tracking of beta-amyloid protein accumulations in 3D two-photon microscopy.

In biology, the monitoring of cell migration and proliferation is fundamental to many research endeavors and requires the tracking of mitotic events. [19] approach this using a deep learning based E2E method combining a CNN for the spatial segmentation and a Long-Short-Term-Memory (LSTM) architecture for the temporal tracking of the cells and their mitotic events. [20] uses 2D Unet extended to a third temporal dimension to track cell positions and motion fields. [12] propose a Graph neural network based method for tracking the cell positions and constructing lineage trees. The cell positions are encoded as node and their association as edges in a graph. An edge classifier predicts the active edges in the graph thereby computing the position tracking and the lineage tree. [21] employ a TBD method using thresholding and Watershed segmentation algorithm for the nuclei segmentation of cancer cells and their subsequent temporal association. Similar to our work, [22] creates a morphogenetic graph to track morphological changes of lesions over time in 3D CT scans.

While most of these methods are domain-specific and tightly couple the detection and the tracking of the instances, we propose a standalone tracking framework that aligns with the TBD paradigm and can be used as post-processing tracking step to any domain-specific instance segmentation algorithm.

## Implementation

**Fig. 2:**
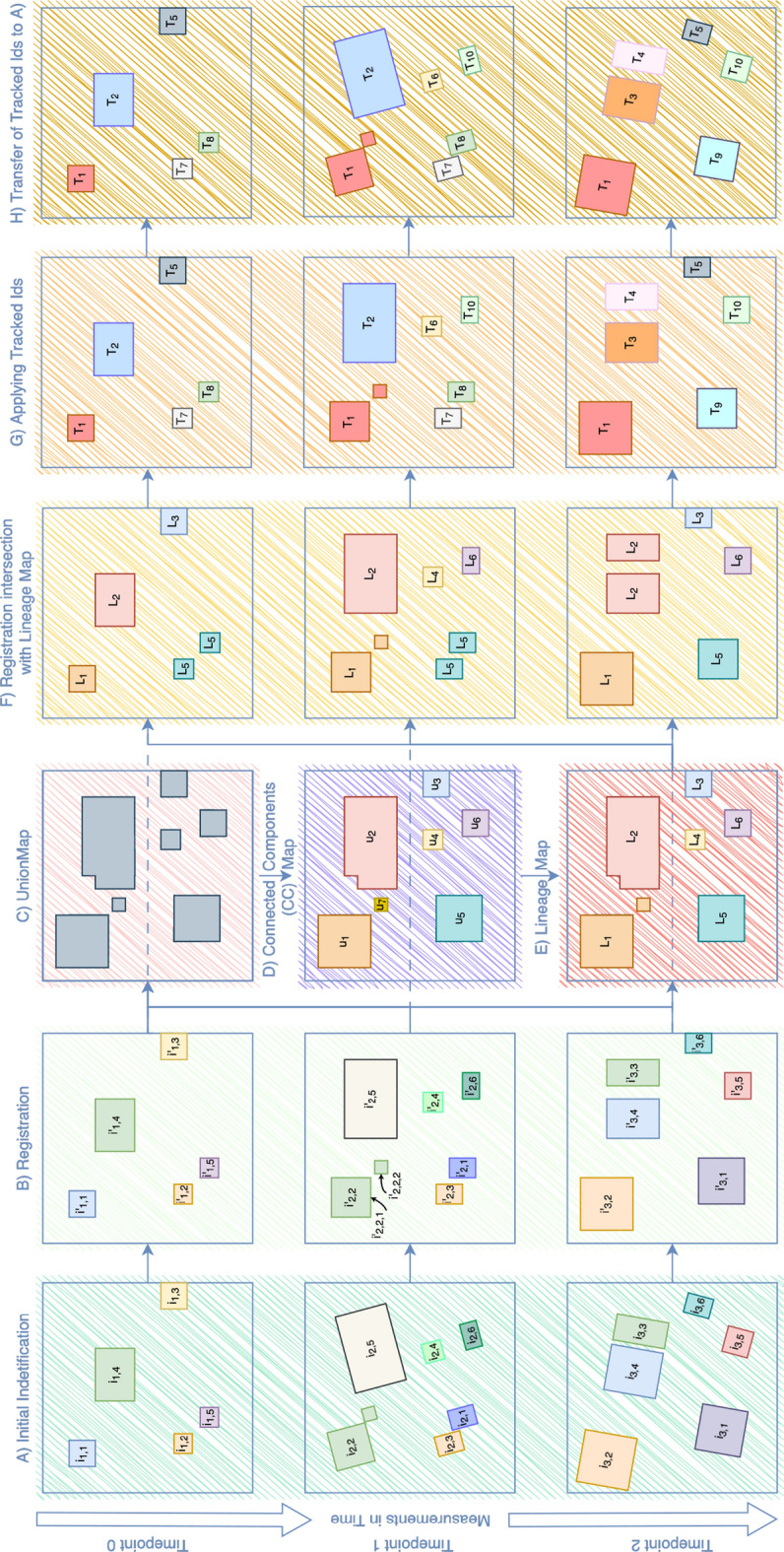
This figure illustrates the central mechanics of the tracking algorithm for scenario S1 for 2D images (similar for 3D)

The primary goal of our instance tracking algorithm is to accurately track the object instances over time and determine the lineage trees. However, when applied to real biological or medical longitudinal data, instance tracking presents numerous challenges, including image misalignment, tissue deformation or sparse temporal resolution of the time-series. Furthermore, the algorithm must often detect various morphogenetic events, such as the emergence of new instances (e.g., metastases), disappearance (e.g., cell death), splitting (e.g., cell division), and the merging of instances (e.g., fusion of molecular accumulations). To address these challenges, our method is structured as a multi-stage sequential pipeline:

- *Input* instance segmentations of the instances for each frame
- *Image Registration* solve camera misalignment and individual instance movement.
- *Union Map* overlap-based maps to determine temporal association of instances across the timeseries.
- *Lineage Map* resolves image registration artifacts, creates the morphological lineage tree and attributes a linage to each instance segmentation.
- *Instance Tracking* assigns temporally-consistent instance ids based on the morphological lineage graphs.
- *Output* lineage IDs and tracked IDs transferred to the original segmentations, and the lineage trees.

### Tracking under scenario S1

**Fig. 3:**
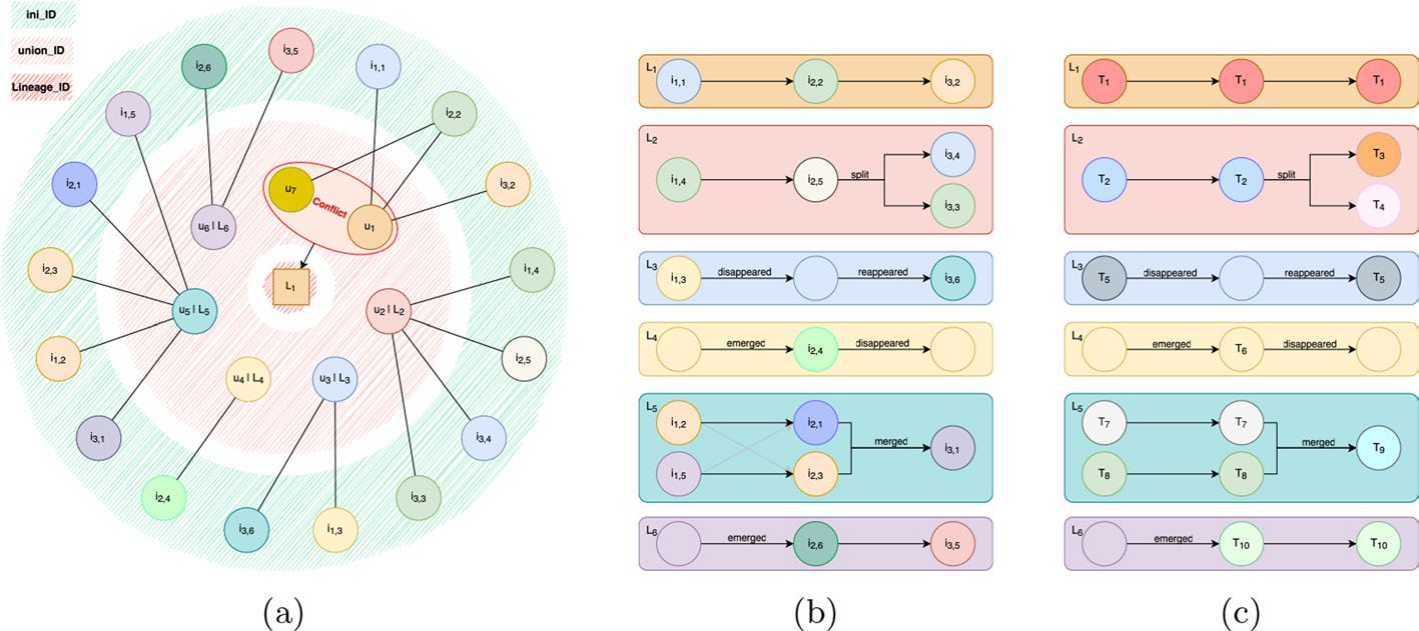
**a** The conflict resolution algorithm illustrated for instances from Fig. 2. **b** The morphogenetic graph can be directly inferred from every connected component in a) when ordering the $i_{t,k}$ by their timepoint *t*. **c)** Tracked_IDs after generating temporally consistent identifiers based on the lineage trees and morphological changes

For scenario S1, we assume the objects of interest to be static relative to each other while the whole ROI can undergo strong changes. If given binary segmentation inputs (e.g. generated by simple thresholding methods) of each of the *T* frames $F_t$, our method begins by conducting a connected component analysis using 26-connectivity for 3D images and a 8-connectivity for 2D images on the binary segmented structures for each timepoint separately, see Fig. 2 A. If the input is already an instance segmentation, this step is omitted. This process identifies $N_t$ distinct instances $I_t=\{i_{t,1}, i_{t,1}, \dots , i_{t,N_t}\}$ for each timepoint *t*. Each instance in $I_t$ has a binary mask $Mask(i_{t,k})$ in the image domain $\Omega$. As these initial identifiers $i_{t,k}$ are calculated per measurement, they are not consistent over the time-series, leading to the same observed object of interest **(OI)** having different identifiers at different timepoints, e.g. if $i_{t,a}$ and $i_{t+1,b}$ are the observations of *OI* at timepoints *t* and $t+1$ it can be that $a\ne b$. This initial instance identification is crucial for detecting morphogenetic events in the lineage trees as shown in Fig. 3b. For example, in Fig. 2, the final lineage identifier $L_2$ (red object in panel *F*) consists of a single initial identifier $i_{2,5}$ in the second measurement and $i_{3,4}$ and $i_{3,3}$ in the third measurement (in panel *A* on the left), indicating that a splitting event occurred.

Tissue movement and misalignment between sequential measurements, which are common in longitudinal and temporally-sparse biological imaging, pose significant challenges for overlap-based tracking algorithms. To address this, our method performs affine image registration of every frame to the first frame in the time series $F_{t\rightarrow r}$ with $r=0$, resulting in the transformed instances $i'_{t,k}$ (Fig. 2 B). This registration transformation can be calculated either on the raw images or the binary segmented structures. In our case, we used the raw microscopy images with subsequent application of the transformation matrix to the instance segmentations using nearest-neighbor interpolation. Alternatively, the registration could be directly performed on the segmentations if sufficient instances serving as landmarks are visible in each segmentation. We opted for the former approach because our method was applied in biological settings where occasionally objects of interest were not yet present in the first measurement of the time series. Since the tracking algorithm requires the images to be aligned, its performance is highly dependent on the accuracy of these registrations. All the registrations are calculated using the cpu-based fast registration tool Greedy [23].

Following the spatial alignment of the measurements, the affine registered instance segmentations are binarized and combined into a single view, referred to as the union-map, using a logical union operator $\bigcup ^T_{t=0}\bigcup ^{N_t}_{k=1}Mask(i'_{t,k})$ (Fig. 2 C). Objects from different measurements in the time series that overlap in the union are considered to be the same observed instance, as they are spatially aligned. A second connected component analysis is then performed on the union-map to derive a new set of overlap-based identifiers (union-ids) establishing a relation between the instances across the the timeseries $\Theta =\{u_1,u_2, \dots , u_m\}$ with $u_m \in \mathbb {N}$ $\forall m=1,2,\dots ,M$. However, it can happen that a single instance $i_{t,k}$ is split apart due to small connections breaking during registration with nearest neighbor interpolation, resulting in multiple disconnected sub-instances $i'_{t,k}=\bigcup ^{n_{t,k}}_{l=1}i'_{t,k,l}$. This results in a conflicting mapping from $i_{t,k}$ to $u_m \in \Theta$, where the registered instance $i'_{t,k}$ is mapped onto multiple union-ids, which is not permissible. This is illustrated in Fig. 2 where instance $i_{2,2}$ is split into two separated components from *A* to *B*, $i'_{2,2,1}$ and $i'_{2,2,2}$, resulting in the mapping to multiple union-ids, $u_1$ and $u_7$, in *D*. Therefore, we need to perform an additional step of conflict resolution where each $i_{t,k}$ is mapped to a unique temporally-consistent identifier (Fig. 2 E).

As illustrated in Fig. 3a, the conflict resolution algorithm is a graph-based adjacency algorithm on the mapping from the temporally-inconsistent initial identifiers $I=\bigcup ^T_{t=0}I_t$ to the temporally-consistent, but potentially-conflicted, union-ids $\Theta$. This mapping can easily be calculated by intersecting each aligned instance segmentation $Mask(i'_{t,k})$ (*B* in Fig. 2) with the connected component map **(CC-map)** (*D* in Fig. 2). In a conflict-free graph, every $i_{t,k}$ would be connected to a single union-id. For example, the adjacency of the initial instances $i_{1,2}$, $i_{1,5}$, $i_{2,3}$, $i_{2,1}$, $i_{3,1}$ and the union-id $u_5$ is a valid mapping. In contrast, if more than one union-id $u_m$ are connected to the same $i_{t,k}$, the union-ids are conflicting, e.g. the adjacency of the instances $i_{1,1}$, $i_{2,2}$, $i_{3,2}$ and the union-ids $u_1$ and $u_7$ is an invalid mapping. These conflicts can be solved by simply remapping every connected group in the graph to a lineage identifier $L_q$ (lineage-id). This process can be streamlined by simply assigning each connect group of union-ids and initial-ids to a single lineage-id. The lineage-ids are generated by indexing the connected groups, ensuring a unique label without ambiguity.

As shown in Fig. 3b, the morphological lineage graphs can be constructed for each lineage $L_q$ by ordering the associated $i_{t,k}$ based on their timepoints *t*. If multiple initial-ids at timepoint *t* are followed by multiple initial-ids at timepoint $t+1$ in the lineage graph, the branching is established by considering the branch with the highest intersection-over-union **(IoU)** score to be the correct branch, see lineage $L_5$ in Fig. 3c. Split and merges can be detected by a varying number of $i_{t,k}$ from one measurement to the next. Disappearance and reappearance can be tracked as all frames of the time series are registered into the same space. The emergence and vanishing of objects can be deducted from the first and last measurement of that instances in the time series.

Temporally-consistent tracking-ids can be created by iterating through the morphological acyclic directed graphs. Starting from the root of each graph, a unique tracked instance id $T_f$ is generated for the first measured objects of each tree and propagated until a split or a merge occurs. If merging or splitting objects are not permissible given the nature of the measured biological system, we can enforce the resolution of such events using the measured IoU between associated instances, e.g. cells are not allowed to merge. The resulting objects from the morphological change are give a new tracked-id, which is again propagated until the next split or merge involving these instances occurs, see Fig. 3c.

Finally, we apply the $i_{t,k}$-to-$L_q$ and the $i_{t,k}$-to-$T_f$ mappings to the original unregistered images, enabling accurate instance tracking in the native image space and preserving both spatial context and anatomical fidelity, as shown in G and H in Fig. 2 for the tracked-ids. This pipeline ensures robust and consistent instance identification across multiple images, even in the presence of image registration imperfections and complex tissue dynamics.

### Tracking under scenario S2

In S2 scenarios, we assume that objects of interest can move significantly and independently of each other over the time series. However, the sampling frequency of the measurements should be high enough so that each object of interest only moves slightly from one time step to the other. As the nature of the movement differs, aligning all the measurements to the first measurement would result in mismatches of the objects as one object can occupy the same spatial location as an other object at a different point in time. The constraint that the objects in the same space in the union-map are the same observed object would be violated.

To track instances accurately in scenario S2, we need to register each measurement to its preceding measurement $F_{t \rightarrow t-1}$ assuming subtle and independent movement of the objects of interest. Subsequently, we create a union-map for each pair of sequential measurements in order to associate objects of interest of each measurement to the instances of the previous measurement. As for S1, we run the connected component analysis to get a CC-map for each of the union-maps. This process links the initial-id $i_{t,k}$ of an observed instance to the initial-id from the preceding and the consecutive measurement $i_{t\pm 1,k}$ forming a chain of initial-ids and union-ids. While the graph-based conflict resolution algorithm was previously only used to correct for interpolation artifacts and the lineage tree generation, it can also resolve the initial and union identifier chains and assign these connected groups in the conflict graph to a single conflict-free identifier. The main advantage is that this method can handle highly dynamic environments. In Scenario S1, temporal associations between individual instance segmentations are established via a union map constructed over the entire time series. This global union implicitly acts as a gap-closing mechanism, enabling the tracking of instances that temporarily disappear and reappear between measurements. In contrast, Scenario S2 relies on pairwise union maps between consecutive frames *t* and $t+1$, which precludes the detection of disappearing and reappearing instances. This constitutes a current limitation of the method, but it could in principle be addressed by extending the pairwise association to span *n* consecutive frames, thereby introducing an *n*-frame gap-closing mechanism, however this extension is left for future work.

## Results

We evaluate the algorithm qualitatively and quantitatively for S1 by tracking Beta-amyloid protein accumulations in 3D in-vivo photon fluorescence microscopy imaging. For S2, we evaluate the performance of the tracking algorithm quantitatively on a diverse set of 2D cell tracking datasets.

### Results on S1

We evaluate the instance tracking algorithm under Scenario S1 by monitoring 3D beta-amyloid accumulations in longitudinal in-vivo two-photon fluorescence microscopy imaging. This dataset is temporally sparse with up to 10 measurements per ROI sampled over a period of 3 months. Due to variations in the imaging setup, such as ROI misalignment, microscope calibration drift, and deformation of the living tissue based on external and biological factors, the dataset presents a challenging setup representative of many research scenarios.

**Fig. 4:**
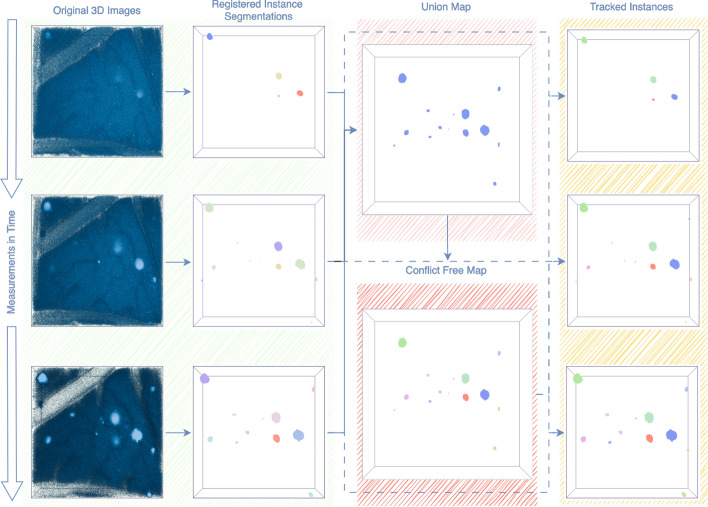
This figure illustrates the tracking algorithm on real biological data, with time-series measurements ordered from top to bottom

As illustrated in Fig. 4, the preprocessing pipeline first produces temporally inconsistent morphological IDs, represented by varying colors of the same components over time. The aligned segmentations are subsequently merged into a single view using a logical union operator, followed by connected component analysis and the conflict resolution algorithm to produce unique and temporally consistent IDs. These are mapped back onto the initial instance segmentations, allowing the determination of when an instance newly appears, disappears, splits, or results from a merging event. The protein accumulations are tracked accurately as indicated by the consistent identification and coloring of each accumulation over time. To quantitatively evaluate tracking performance, we manually annotated a single time series with 240 lineages, containing 825 segmented protein accumulations, varying strongly in volume. The segmentation serving as the basis for manual temporal lineage annotation is identical to the segmentation provided as input to MOLT, ensuring that all nodes in both the ground truth and predicted lineage graphs correspond to the same set of 825 accumulations. Consequently, the total number of nodes is fixed and identical across both graphs. Only the total number of lineages and the assignment of individual accumulations to lineages can differ between prediction and ground truth. For all protein accumulations that interacted over time through fusion or fission events, we assigned a unique lineage identifier. The matching between ground truth and predicted lineages is determined using the Hungarian algorithm [24], with the Intersection over Union (IoU) of the lineage graphs as the cost function. The predicted lineages were compared against the manually annotated ground truth using the Lineage Reconstruction metric (LNR), defined as:

1 $$\begin{aligned} LNR = \max \left( 0,\ 1 - \frac{N_{unmatched} + N_{\text {misassigned}}}{|\mathcal {L}_{gt}| + N_{\text {instances}}}\right) \end{aligned}$$

where $\mathcal {L}_{gt}$ denote the sets of ground truth lineages. $N_{unmatched}$ is the number of unmatched lineages between ground truth and predicted after using the Hungarian algorithm. $N_{\text {misassigned}}$ is the number of instances assigned to an incorrect lineage, and $N_{\text {instances}}$ is the total number of segmented instances. The denominator represents the maximum possible number of corrections required to reconstruct the ground truth lineage graph from scratch. LNR yields a score in the range [0, 1], where higher values indicate more accurate lineage reconstruction. MOLT achieves an LNR score of $83.394\%$, demonstrating high accuracy in lineage reconstruction under complex, biologically realistic conditions involving frequent splitting and merging events. $0.91\%$ of the segmented volume have been wrongly assigned, showing that mostly very small protein aggregations have been assigned to the wrong lineage. For these aggregations, the registration failed to compensate the tissue movement, which was stronger than their physical size, resulting a fragmented union map and multiple predicted lineages.

### Results on S2

To illustrate S2, we utilize three diverse datasets to quantitatively evaluate the performance of the tracking algorithm in S2 scenarios. Fluo-N2DH-GOWT1 [25] features observed GFP-GOWT1 mouse embryonic stem cells at 0.240 x 0.240 microns per pixel resolution and one frame per 5 minutes temporal resolution. Fluo-N2DL-HeLa [26] observes HeLa cells stably expressing H2b-GFP at a 0.645 x 0.645 microns per pixel resolution and one frame per 30 minutes. The final most-challenging dataset, PhC-C2DL-PSC[27], features pancreatic stem cells on a polystyrene substrate at a spatial resolution of 1.6 x 1.6 micros per pixel and a temporal resolution of 10 min per frame. The datasets provide computer-generated silver ground truths with accurate cell segmentations if visible in the frame and manual gold ground truth where the cells are only indicated by non-pixel-accurate annotations also present if the cell is not visible, but present in a given timepoint.

We evaluate tracking performance across two types of input segmentations. First, the silver ground truth segmentations (ST) provided by the Cell Tracking Challenge serve as a high-quality reference input. Second, to evaluate robustness under realistic conditions, we generate automated segmentations using CellPose-SAM (CP-Sam) [9], a generalist microscopy segmentation model, which introduces typical upstream segmentation errors such as missing cells, boundary inaccuracies, and over-segmentation. MOLT is applied as a post-processing tracker on both segmentation inputs.

Tracking performance is evaluated using the Tracking Accuracy (TRA) [29] metric from the Cell Tracking Challenge, which evaluates an algorithm’s capability to correctly identify and link objects across consecutive frames by measuring the disparity between the acyclic oriented graph predicted by the algorithm and the ground truth reference graph. Mathematically, the TRA score is formulated as a normalized version of the Acyclic Oriented Graph Matching (AOGM) metric:

2 $$\begin{aligned} \textrm{TRA} = 1 - \frac{\min (\textrm{AOGM},\ \textrm{AOGM}_0)}{\textrm{AOGM}_0} \end{aligned}$$

where $\textrm{AOGM}$ denotes the transformation cost required to convert the predicted graph into the true reference graph, and $\textrm{AOGM}_0$ represents the baseline cost of constructing the reference graph entirely from an empty state. The inclusion of the minimum operator guarantees that the TRA score remains within [0, 1], where higher values indicate more accurate tracking.

MOLT is benchmarked against three training-free TBD baselines integrated into TrackMate [28]: the LAP Tracker, the Overlap Tracker, and the Advanced Kalman Tracker. These methods were selected as they represent a diverse set of tracking mechanisms, including overlap-based, distance-based, and motion-model-based approaches. It should be noted that these baselines cannot be applied to the S1 protein aggregation dataset, as their underlying topological assumptions of discrete object permanence and tree-like lineage structures are fundamentally violated by the fusion and fission dynamics of protein accumulations. As shown in Table 1, MOLT outperforms all three TrackMate baselines on two of the three datasets across both input segmentation types and achieves competitive performance on the third. The comparatively lower performance on the PhC-C2DL-PSC dataset can be attributed to the higher proportion of missing cell segmentations in this dataset. As discussed in “Tracking under scenario S2” section, Scenario S2 relies on pairwise union maps between consecutive frames and does not support gap closing. Consequently, missing segmentations at intermediate time steps break the pairwise association chain, preventing the correct re-identification of instances upon their reappearance and resulting in fragmented tracklets.

**Table 1:** Comparison of methods across datasets and annotation types using the TRA metric. Bold numbers indicate the best tracking performance for each dataset and annotation type (column).

Method	Fluo-N2DH-GOWT1		Fluo-N2DL-HeLa		PhC-C2DL-PSC	
	ST	CP-Sam	ST	CP-Sam	ST	CP-Sam
Ours	**95.287**	**96.145**	**99.330**	**94.143**	96.797	92.618
Advanced Kalman	94.998	96.002	99.123	94.063	98.289	95.117
LAP Tracker	94.986	95.926	99.130	94.052	**98.813**	95.382
Overlap	94.998	96.054	97.927	92.705	98.714	**95.403**

## Conclusion

The proposed method is an overlap- and lineage-based tracking algorithm, employing a novel conflict resolution procedure to correct registration and tracking errors. It serves as a post-processing step to the instance segmentation in TBD pipelines across various domains. Being training-free, it offers versatility and can be applied to diverse tracking tasks without cost-intensive annotated datasets. The results demonstrate MOLT’s ability to handle scenarios involving frame misalignment, deformable tissues, multiple hundreds of moving objects, and frequent morphological changes. The method returns the tracked instance segmentations together with their lineage information and is readily available as an easy-to-install docker container to facilitate reproducibility.

## Data Availability

The data for the cell tracking experiments can be downloaded from the cell-tracking-challenge website (https://celltrackingchallenge.net/2d-datasets/). The Beta-Amyloid tracking data will be made available after completion of ongoing investigations into the dynamics of the biological systems, expected within 12 months of publication.

## Abbreviations

MOT: Multi-object tracking
TBD: Tracking-by-detection
E2E: End-to-end
S1: No relative movement between objects scenario
S2: Strong relative movement between objects scenario
ROI: Region of interest
CNN: Convolutional neural network
LSTM: Long-short-term-memory
OI: Object of interest
CC-map: Connected component maps
IoU: Intersection-over-Union
IDF1: Identification F1-score
